# Comparison of Cross-Pin Versus Cortical Button Femoral Fixation in Anterior Cruciate Ligament Reconstruction With Hamstrings Autograft: A Long-Term Clinical Study and Review of the Literature

**DOI:** 10.7759/cureus.57928

**Published:** 2024-04-09

**Authors:** Byron Chalidis, Charalampos Pitsilos, Charalampos Pavlopoulos, Polychronis Papadopoulos, Ioannis Gigis, Periklis Papadopoulos

**Affiliations:** 1 1st Orthopaedic Department, Papanikolaou Hospital, Aristotle University of Thessaloniki, Thessaloniki, GRC; 2 2nd Orthopaedic Department, Gennimatas Hospital, Aristotle University of Thessaloniki, Thessaloniki, GRC

**Keywords:** cortical button, endobutton, transfemoral cross-pin, hamstrings autograft, anterior cruciate ligament reconstruction, anterior cruciate ligament

## Abstract

Background

Anterior cruciate ligament reconstruction (ACLR) is a common operative procedure and many options regarding the type of the selected graft and fixation technique have been described to date. Although many studies have addressed the issue of the optimal femoral fixation device during ACLR with a hamstring tendon (HT) autograft, no clear evidence to indicate one technique over another has been found.

Objective

The purpose of this study was to compare the long-term postoperative outcomes and complication rates between transfemoral Cross-pin (CP) and Endobutton-Cortical Button (CB) fixation techniques in patients undergoing ACLR with an HT autograft.

Methods

One hundred and seven consecutive patients underwent ACLR by using a quadruple HT autograft that was stabilized with either a CP (CP Group: 52 patients) or a CB (CB Group: 55 patients) fixation technique. The Lachman test (LT), the Pivot-shift test (PST), the side-to-side difference in anterior translation of the tibia, the International Knee Documentation Committee (IKDC), and the Lysholm knee scoring systems were evaluated before surgery and during long-term follow up. The femoral and tibial tunnel diameter was measured in the anteroposterior (AP) and lateral radiographs after surgery and at the final follow-up. A review of the literature was also carried out to identify any differences between both techniques.

Results

Study groups were comparable in terms of patient demographics. The mean follow-up was 10.4 ± 1.3 and 10.6 ± 1.3 years in the CP and CB Groups, respectively (p = 0.47). In the CP Group, improvements after surgery in LT and PST from grade 2 (n=34) or 3 (n=18) to grade 0 (n = 41) or 1 (n = 11) and from grade 2 (n=36) or 3 (n = 16) to grade 0 (n = 44) or 1 (n = 8), respectively, were observed. In the CB Group, similar improvements in LT and PST scores from grade 2 (n = 40) or 3 (n = 15) to grade 0 (n = 46) or 1 (n = 9) and from grade 2 (n = 41) or 3 (n = 14) to grade 0 (n = 47) or 1 (n = 8), respectively, were observed. However, no differences between the groups (p = 0.53 for LT and p = 0.90 for PST) were noted. The mean Lysholm scores were 89.7 ± 6.8 and 90.2 ± 7.2 in the CP and CB groups, respectively (p = 0.59). Side-to-side difference improved from 9.1 ± 2.8 to 1.7 ± 1.5 mm and from 8.6 ± 2.5 to 1.6 ± 1.4 mm in the CP and CB groups, respectively (p = 0.89 between groups). According to IKDC grades, 92.1% and 91.4% of knees in the CP and CB groups, respectively were reported to be Grade A (Normal) or B (Nearly Normal) with a p = 0.7. Femoral and tibial tunnel widening was found in the last follow-up in both groups. However, there was no difference in the degree of tunnel widening among the two techniques. With respect to LT, PST, anterior drawer test, and IKDC score, none of the 15 published comparative studies demonstrated any significant differences between the two techniques and only one study detected a difference regarding the Lysholm score in favor of CP fixation.

Conclusion

In the long term, both CB and CP femoral stabilization techniques were shown to be associated with similar functional outcomes and low complication rates. Further large multicenter random clinical trials are still required to identify the most effective method of femoral fixation for HT autograft during ACLR surgery.

## Introduction

Different options regarding graft selection and femoral fixation technique have been described so far for anterior cruciate ligament reconstruction (ACLR) [1]. The most common autograft options include the hamstring tendon (HT) and the patella tendon-bone (PTB) grafts. Harvest of HT does not compromise the knee extensor mechanism and it is associated with less donor site pain and lower rates of patellofemoral symptoms and flexion contracture compared to PTB [2]. Although both options offer adequate strength for early mobilization and rehabilitation, the tendon-to-bone incorporation process of HT is significantly slower than the bone-to-bone healing of PTB grafts [3]. The whole process for HT grafts can take up to 12 weeks and may lead to bone tunnel widening (BTW) and impairment of initial knee stability and function [4].

The HT graft is the strongest and stiffest autograft available for biological ACLR with an average ultimate failure load of 4200 N, which exceeds that of native anterior cruciate ligament (ACL) (1725 to 2160 N). However, the fixation interface is the weakest link in the chain, and therefore secure graft fixation is critical during the first weeks after ACLR to withstand the forces on the graft and offer knee stability until graft incorporation and healing. The type of femoral fixation technique has been considered an independent variable for a successful outcome but so far, the optimal construct for stabilizing the graft in the femoral tunnel has not been established. Currently, there are three main options for femoral HT graft fixation: 1) intraosseous interference screws (compression) 2) transfemoral cross-pin (CP) (cortical-cancellous suspension), and 3) cortical buttons (CB) (suspension). Interference screw fixation may cause HT graft laceration from the threads of the screw, loss of pull-out strength, and increase the difficulty of potential revision surgery. The application of suspension devices such as CP and CB can overcome these limitations and avoid early graft failure. Furthermore, they have shown higher maximum failure load and superior biomechanical properties compared to screw fixation [5].

CP fixation system consists usually of two trans-tunnel pins of polylactic acid that fix the HT graft into the femoral tunnel. As its position is closer to the articular surface than the CB, it may decrease graft mobility inside the osseous tunnel. On the other hand, its insertion can cause irritation of the iliotibial band and early implant removal [6]. CB stabilization systems contain a suture loop and a titanium metal plate and have become very popular due to their stability. In this type of fixation, resistance is distributed across the surface between the bone and the device. However, they allow greater movement of the tendon graft within the bone tunnel and their application may cause damage to the adjacent cortical bone [7].

So far, the clinical and biomechanical studies as well as the published meta-analyses and systematic reviews have demonstrated varying results regarding the efficacy of femoral fixation devices [8]. The purpose of this retrospective study was to compare the long-term clinical outcome and complication rates between transfemoral CP fixation over CB during ACLR with HT autograft. A review of the literature was also carried out to identify any differences between both techniques.

## Materials and methods

The study was reviewed and approved by the Institutional Review Board. It is a clinical series of 107 consecutive adult patients (above 18 years of age) who underwent arthroscopically assisted primary ACLR with a quadruple gracilis and semitendinosus autograft from 2008 to 2013. Patients with multiligamentous injuries or menisci tears needing repair were excluded for further evaluation. Furthermore, patients with findings of joint degeneration and osteoarthritis as well as previous knee operations were not evaluated. The first 52 operations were performed by using the Rigidfix (DePuy Mitek, Raynham, MA) CP system (CP Group), and the next 55 operations with the Endobutton (Smith & Nephew, Andover, MA, USA) CB device (CB Group). The procedures were performed by the same surgeon and according to the ethical standards of the institutional committee on human experimentation and with the Helsinki Declaration of 1975, as revised in 2008. Preoperatively, all patients signed a written informed consent form for the use of their anonymized data for research or clinical trials.

After tourniquet thigh application and arthroscopic assessment of the affected knee joint, the ipsilateral semitendinosus and gracilis, and tendons were harvested via a vertical medial side incision. The medial portal technique was used for anatomic single-bundle ACLR. Femoral and tibial tunnel location was determined according to the native ACL tibial and femoral footprint anatomy and its diameter based on the thickness of the graft. A bioabsorbable interference screws 1 mm wider than the graft and tunnel diameter was used for tibial fixation after cycling loading and manual tension. The Rigidfix transcondylar CP or the Endobutton suspensory extracortical device was utilized for graft femoral fixation. Postoperatively, no brace was applied, and weight-bearing as tolerated with crutches was allowed. Isometric quadriceps exercises were immediately commenced to improve muscle strength. Progressive closed-chain exercises were started at six weeks and open-chain at 12 weeks. Running was permitted after six months and return to contact sports activities was allowed after nine months postoperatively.

**Figure 1:**
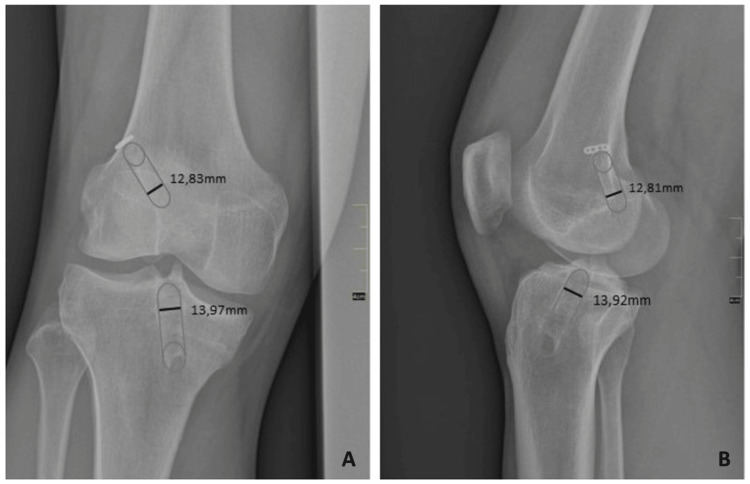
Anteroposterior (A) and lateral (B) knee radiographs showing the method of measuring the femoral and tibial tunnel diameter.

All knees were evaluated preoperatively and at the time of the latest follow-up visit using the International Knee Documentation Committee (IKDC) and Lysholm knee scoring systems. Manual knee laxity was estimated with the Lachman Test (LT) and the Pivot-shift test (PST). The LT was graded as 0 (< 3 mm), 1 (3-5 mm), 2 (6-10 mm), and 3 (> 10 mm). The pivot-shift phenomenon was graded as 0 (equal), 1 (glide), 2 (clunk), and 3 (marked). The femoral and tibial tunnel widening was estimated by comparing immediate postoperative and latest follow-up radiographs. Standard anteroposterior (AP) and lateral knee-joint views were used. The width of the femoral and tibial tunnels was measured digitally just 1 cm from the joint-tunnel interface and perpendicular to the long axis of the tunnels (Figures 1A, 1B). Any postoperative complications or reoperations were also recorded.

Statistical analysis was performed using SPSS software (version 24.0; IBM Corp., Armonk, NY). Continuous data were assessed using the Mann-Whitney U-test. Contingency tables were assessed with the chi-squared test. Comparison between preoperative and postoperative variables among both groups was examined by using the Wilcoxon signed ranks test for continuous data and McNemar’s test for categorical data. The level of significance was set at 5% (p < 0.05).

## Results

Study groups were comparable in terms of patient demographics (Table 1). The mean follow-up was 10.4 ± 1.3 years (range: 8-12 years) in CP Group and 10.6 ± 1.3 years (range: 7-10 years) in CB Group (p=0.47). All patients in both groups experienced significant improvement in terms of IKDC and Lysholm knee scores, LT, and PST (p<0.01 for all parameters).

**Table 1:** Patient’s demographics and results of the clinical examination and patient reported outcome measures CP: Cross-pin; CB: Cortical button; NoP: number of patients; SSD: Side-to-side difference; IKDC: International Knee Documentation Committee ^a^: Mann-Whitney U-test; ^b^: chi-squared test

	Cross-Pin Group (N=52)		Cortical Button Group (N=55)		P-value
Age (years) (range)	26 (18-44)		25 (19-45)		0.65^a^
Gender (M/F)	45/7		47/8		0.87^b^
Follow-up (years)	10.4 ± 1.3		10.6 ± 1.3		0.47^a^
Lysholm Score	60.5 ± 10.8		61.3 ± 12		0.34^a^
SSD (mm)	9.1 ± 2.8		8.6 ± 2.5		0.34^a^
	Type	NoP	Type	NoP	
Lachman Test	0	0	0	0	0.41^b^
	I	0	I	0	
	II	34	II	40	
	III	18	III	15	
Pivot Shift	0	0	0	0	0.54^b^
	1	0	1	0	
	2	36	2	41	
	3	16	3	14	
IKDC Score	A	0	A	0	0.84^b^
	B	0	B	0	
	C	35	C	36	
	D	17	D	19	

In the CP group, the femoral tunnel diameter was increased from 8.85 ± 1.31 mm to 12.74 ± 2.63 mm on AP radiographs (p<0.01) and from 8.91 ± 1.28 mm to 12.32 ± 2.65 mm on lateral radiographs (p<0.01), respectively. Similarly in the CB group, significant enlargement of the femoral tunnel was noticed on both knee views (AP radiographs: from 8.91 ± 1.25 mm to 13.01 ± 2.34 mm (p<0.01). Lateral radiographs: from 9.02 ± 1.32 mm to 12.75 ± 2.05 mm (p<0.01)). However, there was no statistically significant difference between the techniques in both the AP and lateral knee views (p=0.24 and p=0.38, respectively).

In the CP group, the tibial tunnel width was increased from 10.52 ± 1.52 mm to 13.94 ± 1.83 mm on AP radiographs (p<0.01) and from 10.91 ± 1.37 mm to 13.92 ± 2.05 mm on lateral radiographs (p<0.01), respectively. In the CB group, the tibial tunnel diameter was increased from 10.55 ± 1.15 mm to 14.01 ± 1.87 mm (p<0.01) on AP radiographs and from 10.72 ± 1.62 mm to 13.76 ± 2.25 mm (p<0.01) on the lateral radiographs. Nevertheless, no statistically significant difference regarding the postoperative enlargement of tibial tunnel was identified between the two study groups on both the AP and lateral knee views (p=0.64 and p=0.88, respectively).

Complications occurred in three patients. There were two traumatic graft ruptures (1.9%) after two years (CB Group) and four years (CP Group). Both knees required revision surgery using an ipsilateral bone-patella-bone autograft. One patient also at CP Group experienced a superficial infection treated successfully with oral antibiotics only (p=0.48).

## Discussion

The current study revealed similar long-term functional outcomes in patients undergoing ACLR with HT using either the CB or CP devices. All patients faced significant improvement in terms of knee performance and revision surgery was applied after traumatic injury of the operated limb in 1.9% of cases. No implant failure was recorded, and the selected femoral fixation technique did not affect the stability and the long-term survivorship of the graft. Our review of the literature revealed that 15 clinical studies have been published so far comparing the CB and CP methods including 894 knees (455 CB and 439 CP procedures). There were 10 prospective and five retrospective studies enrolling from 22 to 100 patients each and with a follow-up ranging from six to 56 months (Table 2).

**Table 2:** Characteristics of the included studies BIS: bioabsorbable interference screw; CB: cortical button; CP: cross-pin; IS: interference screw; N/A: not available; TT: transtibial technique. AMP: anteromedial portal technique

Authors	Year	Sample (CB/CP)	Type of study	Mean Age (years) (CB/CP)	Gender (M/F)	Follow-up (months)	Time from injury (months)	Meniscal injury (CB/CP)	Femoral Tunnel	Tibial fixation
Fauno and Kaalund [7]	2005	46/41	Prospective	25/26	N/A	12	N/A	N/A	N/A	BIS + washer
Baumfeld et al. [9]	2008	26/20	Retrospective	35.9/36.2		44	N/A	13/14	TT	CB: BIS + staple or screw and washer, CP: Intrafix
Kuskucu et al. [10]	2008	24/32	Prospective	23.9	N/A	26	N/A	N/A	TT	IS + staple
Ibrahim et al. [11]	2009	48/52	Prospective	28	100/0	29	3	28/28	AMP	BIS
Price et al. [6]	2010	13/16	Prospective	26.5/26.3	N/A	24	N/A	10/11	TT	BIS
Sabat et al. [12]	2011	15/15	Prospective	N/A	N/A	22	N/A	N/A	AMP/TT	BIS
Kong et al. [2]	2012	35/56	Retrospective	30.2/31.2	82/9	56	N/A	16/22	TT	CB: BIS + metal screw and washer, CP: Intrafix
Uzumcugil et al. [13]	2012	20/29	Retrospective	28.2/29.8	45/4	30	N/A	N/A	TT	BIS + staple
Eajazi et al. [14]	2013	33/29	Prospective	26.2/23.6	N/A	24	14	N/A	TT	IS
Ibrahim et al. [15]	2015	32/34	Prospective	27	N/A	30	3	12/14	AMP	Bioabsorb IS
Giorgio et al. [16]	2016	20/20	Prospective	34	32/8	6	N/A	N/A	N/A	CB: CB, CP: BIS
Saygi et al. [3]	2016	50/43	Retrospective	26.3/25.9	84/9	41	N/A	19/16	TT	BIS + staple
Srinivas et al. [1]	2016	5/17	Prospective	29.1	N/A	12	N/A	N/A	N/A	IS or Suture Disc
Mousavi et al. [17]	2017	15/15	Prospective	30.5/29.3	25/5	24	20	N/A	N/A	BIS
Lopes et al. [18]	2017	23/20	Retrospective	37.6/30.6	39/4	13	N/A	N/A	AMP	BIS
Total		455/439		28.9		26.2	10			

The available data of these studies could not indicate any clear superiority of one device over another and therefore there is insufficient evidence to recommend a specific femoral fixation technique during ACLR (Table 3).

**Table 3:** Results of the included studies (CP vs CB) CB: cortical button; CP: cross-pin; N/A: not available

Author	LachmanTest	AnteriorDrawer Test	Pivot ShiftTest	KT-1000/ KT-2000	Rolimeter	IKDCScore	LysholmScore	Femoral TunnelWidening	Tibial TunnelWidening	Failure
Fauno and Kaalund [7]	N/A	N/A	N/A	No dif.	N/A	No dif.	N/A	CB	CB	N/A
Baumfeld et al. [9]	N/A	N/A	N/A	No dif.	N/A	No dif.	N/A	CB	No dif.	No dif.
Kuskucu et al. [10]	N/A	N/A	N/A	N/A	N/A	No dif.	No dif.	No dif.	CB	N/A
Ibrahim et al. [11]	No dif.	No dif.	No dif.	No dif.	N/A	No dif.	No dif.	N/A	N/A	N/A
Price et al. [6]	No dif.	No dif.	No dif.	No dif.	N/A	No dif.	N/A	N/A	N/A	No dif.
Sabat et al. [12]	N/A	N/A	N/A	N/A	N/A	No dif.	No dif.	CB	No dif.	N/A
Kong et al. [2]	N/A	N/A	N/A	No dif.	N/A	No dif.	N/A	No dif.	No dif.	N/A
Uzumcugil et al. [13]	No dif.	N/A	No dif.	No dif.	N/A	N/A	No dif.	No dif.	CP	N/A
Eajazi et al. [14]	N/A	N/A	N/A	No dif.	N/A	N/A	CP	N/A	N/A	No dif.
Ibrahim et al. [15]	No dif.	No dif.	No dif.	CP	N/A	No dif.	No dif.	N/A	N/A	N/A
Giorgio et al. [16]	N/A	N/A	N/A	N/A	N/A	N/A	N/A	CB	CB	N/A
Saygi et al. [3]	N/A	N/A	N/A	N/A	No dif.	No dif.	No dif.	No dif.	N/A	N/A
Srinivas et al. [1]	N/A	N/A	N/A	N/A	N/A	N/A	N/A	CP	N/A	N/A
Mousavi et al. [17]	No dif.	No dif.	No dif.	N/A	N/A	No dif.	N/A	N/A	N/A	N/A
Lopes et al. [18]	N/A	N/A	N/A	N/A	No dif.	No dif.	No dif.	CP	N/A	N/A

Biomechanical analysis of CB and CB fixation techniques has shown that both options can offer adequate early stability. Shen et al. [19] in an animal model study using porcine flexor digitorum profundus tendon graft in fresh porchine knees found that both CB and CP devices were equally strong and suitable for early aggressive rehabilitation. In addition, Rodriguez et al. [4] using synthetic grafts found no significant differences in terms of stiffness, failure strength, and elongation to failure before and after cyclic loading between both techniques. On the other hand, Abidin et al. [20] in a biomechanical comparison of the two methods using finite element modeling and analysis reported that CP fixation was associated with better stability and lower stress and strain at fixators, bones, and menisci than the CB fixation. Hexter et al. [21] in a sheep animal study underwent unilateral single-bundle ACLR using a porcine superflexor tendon xenograft. Although at 11 weeks there was no difference in range of motion between the two techniques, there was a better ground reaction force after CB fixation that was associated with earlier functional recovery.

Early instability after ACLR surgery may occur due to flawed surgical technique, inappropriate graft fixation, premature exercise, or inappropriate physiotherapy, whereas delayed instability (beyond one year) is usually a result of repeated trauma of the ACL graft tissue. Therefore, fixation devices play a paramount importance in graft stability during the first weeks after operations since more than 50% of total elongation takes place in the first 20 cycles [4]. Both techniques show different failure patterns. Insufficient femoral fixation of the button or damage of the cortex during drilling or flipping of the implant are the main factors for graft instability in cortical suspension devices. In cortical-cancellous fixation systems, pin breakage, prominence, stress fracture, and migration are potential risks for failure. In addition, posterior transcortical breach and drilling through the anteromedial portal predispose to pin fracture, which may occur in up to 39% of cases. However, this is not always of clinical importance and rarely requires further surgery [22]. 

Postoperative knee laxity is one of the most essential predictors of knee function and has been thoroughly evaluated after ACLR with either the CP or CB fixation techniques. With respect to LT, PST, and anterior drawer tests, none of the published comparative studies demonstrated any statistically significant difference between the two techniques. This was also the conclusion of the meta-analysis of Jiang et al. [8] which included six level I or II RCTs with 445 patients. However, Ibrahim et al. [11,15] found that patients treated with CP had slightly better LT, PST, and anterior drawer test post-operative outcomes but failed to reach statistical significance. On the other hand, Price et al. [6] and Mousavi et al. [17] found marginally better postoperative knee stability in the CB group compared to the CP group, also without any statistical significance. In our study, we did not identify any difference regarding the knee laxity tests between the two techniques after a mean 10-year follow-up. 

The IKDC and the Lysholm score systems have been mainly used for the evaluation of postoperative knee function after ACLR using the CB and CP femoral fixation techniques. From the 16 published comparative trials (including our long-term study), only one demonstrated a difference regarding the Lysholm score in favor of CP fixation [14]. Saccomanno et al. [23] in a systematic review including five Level I and II RCTs with 317 patients did not find any short- to medium-term differences in IKDC and Lysholm scores between patients treated with CB femoral graft fixation and those treated with suspensory transfemoral fixation during ACLR. Furthermore, according to the available data, the rates of postoperative satisfaction and ability to perform sports activities were also comparable between the two groups and only one RCT showed better results after the CB fixation technique [17]. However, the published studies did not include athletes or high-level sports participants, and therefore no definite conclusion can be drawn in this population group regarding the superiority of one technique over the other. 

BTW after ACLR is a relatively early (during the first year) radiologic finding that may jeopardize graft incorporation and durability. However, its appearance has not been clearly proven to be associated with an inferior result [23]. It seems to be an anticipated complication of graft fixation, depending on both mechanical factors, such as fixation method and improper graft placement, and biological factors, such as graft type and synovial fluid propagation. Yue et al. [24] proposed the possible mechanisms of BTW including bone necrosis from drilling, foreign body response, longitudinal and transverse graft motion, elevated inflammatory cytokine levels in synovial fluid during ACLR, stimulation of osteoclasts with concomitant inhibition of osteoblastic activity, cell apoptosis after ACL rupture and microbial colonization. Moreover, drilling the femoral tunnel from the medial portal seems to result in smaller postoperative tunnel enlargements compared to the transtibial technique, while a more anterior and proximal tunnel is associated with greater BTW. This was partially confirmed by Lee et al. [25], who reported that anterior femoral tunnel placement was correlated with increased BTW after two years from ACLR.

Femoral BTW has been also associated with the applied femoral fixation technique and it may be more than 50% in up to 28% of cases. Our review of the literature revealed that the CB was associated with greater BTW in four studies [7,9,12,16] and the CP in two [1,18]. In four studies, no difference was identified [2,3,10,13]. Lee et al. [26] performed a systematic review that included all levels of evidence in studies that reported femoral BTW and compared CB and CP femoral fixation. The authors identified a slightly greater widening of the femoral tunnel when using CB via the transtibial technique than when using transfemoral CP fixation. They advocated that the “bungee and wind-shield wiper effects” of the CB technique might explain the higher incidence of femoral BTW after HT autograft ACLR surgery. We also noticed enlargement of the femoral tunnel aperture after ACLR, but the relevant values were similar in CP and CB fixation groups. In our study, we measured the tunnels width at 1 cm from the articular surface in knee radiographs with a magnification rate of 1:1. Other techniques, such as using the head of the interference screw as a reference point have been also described [27]. 

The incidence of postoperative complications after ACLR is quite low but some of these may be related to the femoral fixation method. Studler et al. [28] in an MRI study found that CP fixation was associated with pin fracture in 17% of cases, migration of fractured pin in 6% of cases, posterior femoral cortex breach in 28% of cases, lateral femoral cortex CP prominence in 3% of cases and partial or complete graft tear in 4% of cases. On the other hand, the application of CB has been correlated with inappropriate intra-tunnel fixation and button migration due to possible soft tissue interposition between the device and the lateral femoral cortex. Ibrahim et al. [11] found a 4% CB migration rate in 50 patients undergoing double-bundle hamstring autograft ACLR. Also, Kong et al. [2] noticed that after four years postoperatively, there was an advancement of osteoarthritis of grade 1 according to the Kellgren-Lawrence scale at 9% and 5% of patients treated with a CP or CB technique, respectively. Grassi et al. [29] in a systematic review observed an incidence of 13.4% of clinical failure and 7.9% of revision rate at 20 years after ACLR. In addition, Eajazi et al. [14] and Baumfeld et al. [9] reported failure rates of 18% and 25% after CB and CP fixation, respectively. Persson et al. [30] in a register-based study of 38.666 patients undergoing primary ACLR with HT, noticed that independently of the type of tibial fixation, the revision rate was reduced by 30% when transfemoral CP was applied instead of CB fixation. In our study, we detected only a rate of 2% of traumatic graft rupture and reoperation in both groups without any difference between the two techniques.

The presented study has certain limitations. Firstly, it represents a single institution’s retrospective study with the inherent limitations of such a design. Secondly, the operations were not carried out in the same period and under identical circumstances. On the other hand, it contains a homogenous group of patients that underwent only ACLR without any concomitant injuries by one surgical team consecutively and using the same knee approach. Furthermore, and according to our knowledge, it contains the largest number of patients with the longest follow-up period in a published series on this subject to date.

## Conclusions

In the long-term, both CB and CP femoral stabilization techniques are associated with significant improvement in knee stability and performance as well as low complication rates. However, the magnitude of functional improvement is similar in both groups and the available data could not indicate any clear superiority of one device over another. Further large multicenter RCTS are still required to identify the most effective method of femoral fixation for HT autograft during ACLR surgery.

## References

[1] Srinivas DK, Kanthila M, Saya RP, Vidyasagar J (2016). Femoral and tibial tunnel widening following anterior cruciate ligament reconstruction using various modalities of fixation: a prospective observational study. J Clin Diagn Res.

[2] Kong CG, In Y, Kim GH, Ahn CY (2012). Cross pins versus Endobutton femoral fixation in hamstring anterior cruciate ligament reconstruction: minimum 4-year follow-up. Knee Surg Relat Res.

[3] Saygi B, Karaman O, Sirin E, Arslan I, Demir A, Oztermeli A (2016). Comparison of different femoral fixation implants and fit techniques for tunnel widening and clinical outcome in ACL reconstruction using hamstring autograft. Arch Orthop Trauma Surg.

[4] Rodríguez C, García TE, Montes S, Rodríguez L, Maestro A (2015). In vitro comparison between cortical and cortico-cancellous femoral suspension devices for anterior cruciate ligament reconstruction: implications for mobilization. Knee Surg Sports Traumatol Arthrosc.

[5] Espejo-Baena A, Ezquerro F, de la Blanca AP, Serrano-Fernandez J, Nadal F, Montañez-Heredia E (2006). Comparison of initial mechanical properties of 4 hamstring graft femoral fixation systems using nonpermanent hardware for anterior cruciate ligament reconstruction: an in vitro animal study. Arthroscopy.

[6] Price R, Stoney J, Brown G (2010). Prospective randomized comparison of endobutton versus cross-pin femoral fixation in hamstring anterior cruciate ligament reconstruction with 2-year follow-up. ANZ J Surg.

[7] Fauno P, Kaalund S (2005). Tunnel widening after hamstring anterior cruciate ligament reconstruction is influenced by the type of graft fixation used: a prospective randomized study. Arthroscopy.

[8] Jiang H, Ma G, Li Q, Hu Y, Li J, Tang X (2018). Cortical button versus cross-pin femoral fixation for hamstring anterior cruciate ligament reconstruction: a meta-analysis of randomized controlled trials. Am J Sports Med.

[9] Baumfeld JA, Diduch DR, Rubino LJ, Hart JA, Miller MD, Barr MS, Hart JM (2008). Tunnel widening following anterior cruciate ligament reconstruction using hamstring autograft: a comparison between double cross-pin and suspensory graft fixation. Knee Surg Sports Traumatol Arthrosc.

[10] Kuskucu SM (2008). Comparison of short-term results of bone tunnel enlargement between EndoButton CL and cross-pin fixation systems after chronic anterior cruciate ligament reconstruction with autologous quadrupled hamstring tendons. J Int Med Res.

[11] Ibrahim SA, Hamido F, Al Misfer AK, Mahgoob A, Ghafar SA, Alhran H (2009). Anterior cruciate ligament reconstruction using autologous hamstring double bundle graft compared with single bundle procedures. J Bone Joint Surg Br.

[12] Sabat D, Kundu K, Arora S, Kumar V (2011). Tunnel widening after anterior cruciate ligament reconstruction: a prospective randomized computed tomography--based study comparing 2 different femoral fixation methods for hamstring graft. Arthroscopy.

[13] Uzumcugil O, Yalcinkaya M, Ozturkmen Y, Dikmen G, Caniklioglu M (2012). Effect of PEEK polymer on tunnel widening after hamstring ACL reconstruction. Orthopedics.

[14] Eajazi A, Madadi F, Madadi F, Boreiri M (2013). Comparison of different methods of femoral fixation anterior cruciate ligament reconstruction. Acta Med Iran.

[15] Ibrahim SA, Abdul Ghafar S, Marwan Y (2015). Intratunnel versus extratunnel autologous hamstring double-bundle graft for anterior cruciate ligament reconstruction: a comparison of 2 femoral fixation procedures. Am J Sports Med.

[16] Giorgio N, Moretti L, Pignataro P, Carrozzo M, Vicenti G, Moretti B (2016). Correlation between fixation systems elasticity and bone tunnel widening after ACL reconstruction. Muscles Ligaments Tendons J.

[17] Mousavi H, Maleki A, Nobakht A (2017). Comparative study after hamstring anterior cruciate ligament reconstruction with Endobutton and Rigidfix: a clinical trial study. Adv Biomed Res.

[18] Lopes OV Jr, de Freitas Spinelli L, Leite LH, Buzzeto BQ, Saggin PR, Kuhn A (2017). Femoral tunnel enlargement after anterior cruciate ligament reconstruction using RigidFix compared with extracortical fixation. Knee Surg Sports Traumatol Arthrosc.

[19] Shen HC, Chang JH, Lee CH, Shen PH, Yeh TT, Wu CC, Kuo CL (2010). Biomechanical comparison of cross-pin and Endobutton-Cl femoral fixation of a flexor tendon graft for anterior cruciate ligament reconstruction-a porcine femur-graft-tibia complex study. J Surg Res.

[20] Zainal Abidin NA, Abdul Wahab AH, Abdul Rahim RA, Abdul Kadir MR, Ramlee MH (2021). Biomechanical analysis of three different types of fixators for anterior cruciate ligament reconstruction via finite element method: a patient-specific study. Med Biol Eng Comput.

[21] Hexter AT, Hing KA, Haddad FS, Blunn G (2020). Decellularized porcine xenograft for anterior cruciate ligament reconstruction: a histological study in sheep comparing cross-pin and cortical suspensory femoral fixation. Bone Joint Res.

[22] Xu Y, Ao Y, Wang J, Yu J, Cui G (2011). Relation of tunnel enlargement and tunnel placement after single-bundle anterior cruciate ligament reconstruction. Arthroscopy.

[23] Saccomanno MF, Shin JJ, Mascarenhas R, Haro M, Verma NN, Cole BJ, Bach BR Jr (2014). Clinical and functional outcomes after anterior cruciate ligament reconstruction using cortical button fixation versus transfemoral suspensory fixation: a systematic review of randomized controlled trials. Arthroscopy.

[24] Yue L, DeFroda SF, Sullivan K, Garcia D, Owens BD (2020). Mechanisms of bone tunnel enlargement following anterior cruciate ligament reconstruction. JBJS Rev.

[25] Lee SS, Kim IS, Shin TS, Lee J, Lee DH (2023). Femoral tunnel position affects postoperative femoral tunnel widening after anterior cruciate ligament reconstruction with tibialis anterior allograft. J Clin Med.

[26] Lee DH, Son DW, Seo YR, Lee IG (2020). Comparison of femoral tunnel widening after anterior cruciate ligament reconstruction using cortical button fixation versus transfemoral cross-pin fixation: a systematic review and meta-analysis. Knee Surg Relat Res.

[27] Karikis I, Ejerhed L, Sernert N, Rostgård-Christensen L, Kartus J (2017). Radiographic tibial Tunnel assessment after anterior cruciate ligament reconstruction using hamstring tendon autografts and biocomposite screws: a prospective study with 5-year follow-up. Arthroscopy.

[28] Studler U, White LM, Naraghi AM, Tomlinson G, Kunz M, Kahn G, Marks P (2010). Anterior cruciate ligament reconstruction by using bioabsorbable femoral cross pins: MR imaging findings at follow-up and comparison with clinical findings. Radiology.

[29] Grassi A, Pizza N, Al-Zu'bi BB, Fabbro GD, Lucidi GA, Zaffagnini S (2022). Clinical outcomes and osteoarthritis at very long-term follow-up after ACL reconstruction: a systematic review and meta-analysis. Orthop J Sports Med.

[30] Persson A, Gifstad T, Lind M (2018). Graft fixation influences revision risk after ACL reconstruction with hamstring tendon autografts. Acta Orthop.

